# *BRCA1 *promoter methylation in peripheral blood DNA of mutation negative familial breast cancer patients with a *BRCA1 *tumour phenotype

**DOI:** 10.1186/bcr1858

**Published:** 2008-02-12

**Authors:** Cameron Snell, Michael Krypuy, Ee Ming Wong, kConFab investigators, Maurice B Loughrey, Alexander Dobrovic

**Affiliations:** 1 Molecular Pathology Research and Development Laboratory, Department of Pathology, Peter MacCallum Cancer Centre, Locked Bag 1, A'Beckett St, Melbourne, Victoria 8006, Australia; 2 Department of Pathology, The University of Melbourne, Parkville, Victoria, 3010, Australia; 3 Department of Pathology, Royal Group of Hospitals, Belfast, Northern Ireland

## Abstract

**Introduction:**

Individuals with germline mutations in the *BRCA1 *gene have an elevated risk of developing breast cancer, and often display characteristic clinicopathological features. We hypothesised that inactivation of *BRCA1 *by promoter methylation could occur as a germline or an early somatic event that predisposes to breast cancer with the phenotype normally associated with *BRCA1 *germline mutation.

**Methods:**

We examined seven cases from breast-ovarian cancer families with tumours that showed *BRCA1*-like pathology but did not have detectable *BRCA1 *or *BRCA2 *germline mutations present. Methylation levels were tested by several quantitative techniques including MethyLight, methylation-sensitive high resolution melting (MS-HRM) and a newly developed digital MS-HRM assay.

**Results:**

In one patient, methylation of 10% of the *BRCA1 *alleles was detected in the peripheral blood DNA, consistent with 20% of cells having one methylated allele. Buccal mucosa DNA from this individual displayed approximately 5% *BRCA1 *methylation. In two other patients, methylation of *BRCA1 *was detected in the peripheral blood at significantly lower but still readily detectable levels (approximately 1%). Tumour DNAs from these three patients were heavily methylated at *BRCA1*. The other patients had no detectable *BRCA1 *methylation in their peripheral blood. One of seven age-matched controls showed extremely low levels of methylation in their peripheral blood (approximately 0.1%).

**Conclusion:**

These results demonstrate that in some cases of breast cancer, low-level promoter methylation of *BRCA1 *occurs in normal tissues of the body and is associated with the development of *BRCA1*-like breast cancer.

## Introduction

In 1994, *BRCA1 *was identified as the first major gene associated with familial breast cancer predisposition [1]. Since then many inactivating mutations in *BRCA1 *have been identified as breast cancer predisposition alleles.

Breast cancers associated with *BRCA1 *mutations often show characteristic histological features including high grade, high mitotic count, solid architecture and prominent lymphocytic infiltrates, all features resembling so-called medullary cancer [2-4]. *BRCA1*-associated tumours are usually negative by immunohistochemistry for the oestrogen receptor (ER), the progesterone receptor (PR) and HER2 [5-7]. However, the majority of breast cancers that exhibit a *BRCA1*-like phenotype do not harbour detectable germline mutations in *BRCA1*. Some of this discordance may be due to epigenetic defects in breast cancer susceptibility genes such as *BRCA1 *contributing to breast cancer predisposition.

Recent reports of somatic methylation (or epimutations) affecting an allele of the *MLH1 *gene in patients with hereditary non-polyposis colorectal cancer indicate inactivation of tumour suppressor genes by promoter methylation can occur early in development or possibly in the germline [8-10]. Allelic methylation is functionally equivalent to a mutation in that loss of activity of the second allele arising from a mutation, loss of heterozygosity or a second methylation event will inactivate the gene.

Methylation of the *BRCA1 *promoter has been shown to occur in approximately 20% of breast cancer patients [11-14]. Sporadic tumours with *BRCA1 *promoter methylation have been reported to be ER and PR negative [13,14], or to display similar pathological features to those of *BRCA1*-mutated hereditary breast cancers [15]. Furthermore, tumours with *BRCA1 *methylation appear to have similar global gene expression profiles to *BRCA1 *mutated tumours [16] and similar genomic copy number profiles [17].

Other authors have claimed that *BRCA1 *methylated tumours have distinct pathologies to those seen in *BRCA1 *mutated tumours [18]. The discrepancy may at least in part be resolved by the hypothesis that the timing of *BRCA1 *methylation will influence tumour phenotype; the earlier in tumorigenesis methylation occurs, the greater the similarity to tumours arising from germline *BRCA1 *mutations. It must also be taken into consideration that while most tumours arising in *BRCA1 *mutation carriers have typical pathology, a sizeable minority do not.

We hypothesised that some individuals are predisposed to develop breast cancer with the features associated with *BRCA1 *mutations because they carry a methylated *BRCA1 *allele in their somatic tissues.

## Materials and methods

### Individuals and study samples

The research was completed in compliance with the Helsinki Declaration. The Ethics of Human Research Committee of Peter MacCallum Cancer Centre approved the study (approval number 02/70). Individuals used in the study were enrolled in the Kathleen Cuningham Consortium for Research in Familial Breast Cancer (kConFab). kConFab identified seven breast cancer cases (KCF1-7) with *BRCA1*-like features for analysis. kConFab provided DNA extracted from peripheral blood leukocytes from each case and DNA from buccal mucosa of patient KCF3. Tumour material from the KCF1, KCF2, KCF3, KCF4 and KCF6 cases was available. Four unstained, formalin-fixed, paraffin-embedded sections from each tumour were provided for analysis. DNA was extracted from the paraffin sections using the DNeasy tissue kit (Qiagen, Hilden, Germany). Normal control DNAs were provided by the Peter MacCallum Cancer Centre tissue bank.

### Methylation analysis

CpGenome Universal Methylated DNA (Chemicon, Millipore, Billerica, MA, USA) was used as the 100% methylated control and DNA extracted from peripheral blood mononuclear cells of normal individuals was used as unmethylated control DNA. Bisulfite modification of DNA was performed with the MethylEasy kit (Human Genetic Signatures, Sydney, Australia) according to the manufacturer's instructions. Methylation standards were constructed by diluting 100% methylated control DNA (bisulfite modified) in a pool of normal DNA (bisulfite modified) at 50%, 25%, 10%, 5% and 1% ratios.

**Figure 1:**
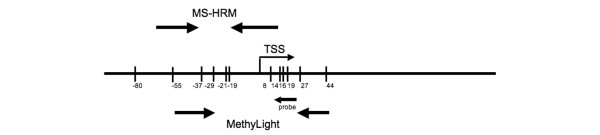
Map of the *BRCA1 *promoter region studied by the MethyLight and MS-HRM assays. The numbering of the promoter is according to that used by Rice *et al*. [24]. TSS denotes the transcription start site. The positions of the primers flanking the MethyLight and MS-HRM amplicons are indicated as well as the position of the MethyLight probe.

Methylation of the *BRCA1 *promoter was assessed using real-time methylation specific PCR (MethyLight) [19] and methylation-sensitive high resolution melting (MS-HRM) [20]. All samples were run in triplicate for each assay. The locations of the MethyLight and MS-HRM primers and probes on the *BRCA1 *promoter are illustrated in Figure 1. The MethyLight assay assessed five CpG sites and the MS-HRM assay assessed four sites. Careful design of each assay was required to avoid amplification from the *BRCA1 *pseudogene. The *HMBS *gene was used as a control for the *BRCA1 *MethyLight assay. Primer sequences are available on request.

Experiments were performed on the RotorGene™ 3000 (MethyLight assays) and RotorGene™ 6000 (MS-HRM assays) instruments (Corbett Research, Sydney, Australia). The MethyLight data was analysed by obtaining the take-off (Ct) and amplification efficiency values for each sample for *BRCA1 *and *HMBS *from the comparative quantitation tab of the RotorGene™ analysis software and comparing them to the values for the 100% methylated DNA according to the Pfaffl method of relative quantification [21].

Digital MS-HRM was developed to confirm the results of the *BRCA1 *MethyLight and MS-HRM assays. Serial 10-fold dilutions of bisulfite modified DNA from the methylation positive samples were made and replicates of each dilution were amplified by MS-HRM. The dilution at which amplification of some replicates dropped out was then chosen for further analysis. Multiple replicate amplifications for the selected dilution were performed and compared to 0% and 100% methylated control DNA. Reactions or 'clones' resembling fully methylated and unmethylated DNA were then used in a second round amplification with M13 tagged primers. The products were then sequenced using M13 primers using the BigDye terminator kit (Applied Biosystems, Foster City, CA, USA).

## Results

We examined the peripheral blood leukocyte DNA of seven breast cancer cases (KCF1 to KCF7) (ages of onset ranging from 35 to 51 years) for *BRCA1 *methylation. Each of these cases, from the kConFab familial cancer repository, had a family history of breast or ovarian cancer with *BRCA1*-like breast pathology as defined by kConFab criteria, but were negative for mutations in the coding regions of *BRCA1 *and *BRCA2*.

We used both real-time methylation specific PCR (MethyLight) [19] and methylation-sensitive high resolution melting (MS-HRM) [20] to assess methylation levels. The primers for each assay spanned the proximal *BRCA1 *promoter region and were carefully designed so that the methylation status of the highly homologous pseudo *BRCA1 *region [22] was not assessed (Figure 1). Three of the individuals showed detectable *BRCA1 *methylation in DNA from their peripheral blood leukocytes, but unexpectedly all showed less than the 50% methylation that would be expected for methylation at one allele in every cell.

**Figure 2:**
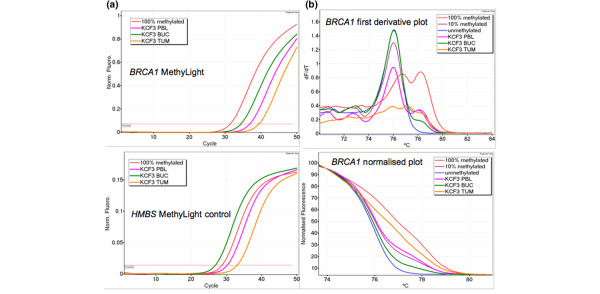
Methylation analysis of *BRCA1 *in KCF3. **(a)** MethyLight results for *BRCA1 *and control gene *HMBS *for samples from KCF3. The *BRCA1 *MethyLight assay indicates the presence of methylated DNA for the peripheral blood leukocyte (PBL), buccal mucosa (BUC) and tumour (TUM) of KCF3. The *HMBS *control gene indicates the amount of bisulfite modified DNA for each sample of KCF3. **(b)** Methylation-sensitive high resolution melting (MS-HRM) results for samples from KCF3. The PBL sample (pink curve) and the BUC sample (green curve) show methylation levels close to the 10% methylated control. The TUM sample (orange curve) shows a much higher level of methylation.

KCF3 was the sample showing the highest level of methylation of *BRCA1*. *BRCA1 *methylation was detected in the peripheral blood DNA at approximately 14% methylated alleles estimated by MethyLight, and 10% methylated alleles estimated by MS-HRM (Figure 2). Buccal mucosal DNA from this patient showed approximately 10% methylated alleles estimated by MethyLight and 5% methylated alleles estimated by MS-HRM. Thus, both methods gave a similar estimate of methylation.

To verify that the deviation from the expected 50% methylation was not due to PCR bias or other artefacts, we developed a third methodology specifically to accurately quantify the levels of methylation. Digital MS-HRM is an adaptation of MS-HRM that enables rapid counting of methylated and unmethylated alleles. The digital approach to quantification has been described previously [23]. However, previous methods have relied on subsequent secondary analysis whereas HRM uses in-tube analysis with a consequent rapid generation of results.

In digital MS-HRM, bisulfite modified DNA is diluted to the point where the individual PCR reactions contained 0, 1 or occasionally 2 amplifiable templates. This eliminates the problem of PCR bias where unmethylated or methylated templates have different amplification efficiencies, hence skewing the actual methylated to unmethylated ratios. Multiple replicates of the diluted sample are amplified by PCR where it is expected that many reactions will not amplify because of the absence of template. The melting profile of each reaction or 'clone' is then used to determine its methylation status. The proportion of methylation in a sample can be readily estimated by comparing the number of reactions with a peak with elevated melting temperature characteristic of methylation, over those with an lower melting temperature unmethylated peak.

**Figure 3:**
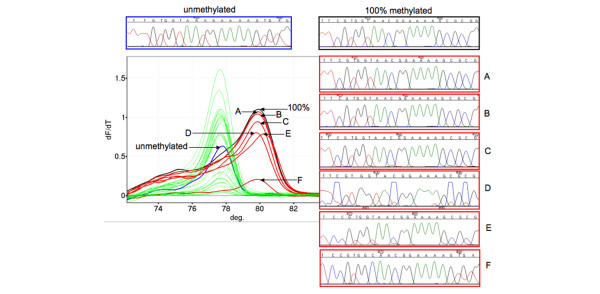
Digital methylation-sensitive high resolution melting (MS-HRM) and sequencing for KCF3 peripheral blood DNA. The blue curve indicates the unmethylated control. The black curve indicates the methylated control. The green curves indicate unmethylated amplicons. The red curves indicate methylated amplicons. Sequencing for the unmethylated and methylated controls are shown above the digital MS-HRM results. Sequences of the indicated red amplicons (lettered) are shown to right of the figure where the letter to the right of the chromatograph corresponds to the curve shown.

Digital MS-HRM was performed on peripheral blood and buccal mucosal DNA from KCF3. For the peripheral blood DNA, 13 of the 107 clones had a methylated peak on melting analysis (Figure 3), equating to 12% *BRCA1 *methylation in the peripheral blood. In the buccal mucosa, 4 of the 69 clones were methylated, equating to 6% methylation. These results confirmed the level of *BRCA1 *methylation estimated by both MethyLight and MS-HRM. When considered at a cellular rather than an allelic level, we estimate that the peripheral blood contains approximately 25% of cells with one methylated *BRCA1 *allele and that the buccal mucosa contains approximately 10% methylated cells.

Representative methylated and unmethylated clones from the digital MS-HRM experiments were sequenced to confirm the methylation status (Figure 3). A stretch of 22 bases containing four CpG sites was analysed. For the peripheral blood DNA of KCF3 (Figure 3), four of the six methylated clones had a cytosine present at all four CpGs, confirming the methylation seen by other methodologies. One clone had cytosines present at all CpGs sites but also had an additional cytosine present at a conversion site where a thymine would be expected, preceding a CpG site. The other non-CpG cytosines were converted indicating that this corresponded to a methylated allele. For one other clone only two of the CpGs were methylated but this may have been due to incomplete conversion.

Patient KCF3 developed two breast cancers in her lifetime, the first at 43 years and the second at 51 years. DNA from the first tumour was available for analysis and both MethyLight and MS-HRM assays indicated that *BRCA1 *methylation was present. Analysis using digital MS-HRM estimated the tumour methylation at 61% (14/23 clones).

We examined the peripheral blood and tumour DNA of KCF3 for methylation at other loci that are commonly methylated in breast cancer to assess whether the observed *BRCA1 *methylation was due to a propensity to methylate at a global level. The samples were screened with MethyLight. None of *RASSF1A, RARB, GSTP1, TWIST, CDH1, HIC1 *and *HIN1 *proved to be methylated (results not shown) indicating that the methylation in this patient seen is not part of an overall increased methylation of CpG islands.

**Figure 4:**
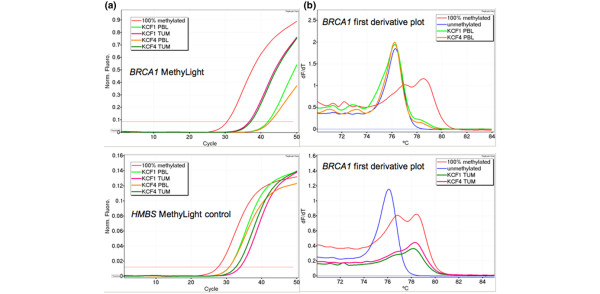
Methylation analysis of *BRCA1 *in KCF1 and KCF4. **(a)** MethyLight results for *BRCA1 *and the control gene *HMBS *for samples from KCF1 and KCF4. The *BRCA1 *MethyLight assay indicates the presence of methylated DNA for the peripheral blood (PBL) and tumour (TUM) for both individuals. **(b)** The methylation-sensitive high resolution melting (MS-HRM) assay also shows the presence of methylation in the peripheral blood and tumour for both individuals.

In two of the remaining cases, KCF1 and KCF4, methylation of *BRCA1 *was detected at approximately 1% in the peripheral blood DNA using MethyLight and MS-HRM (Figure 4). Digital MS-HRM was used to confirm the observed methylation in these samples and 1 of 33 clones for KCF1 and 1 of 44 for KCF4 showed a fully methylated peak. Analysis of tumour material from both of these individuals demonstrated close to 100% methylation using MethyLight and MS-HRM.

Tumour material was available from two of the four patients that did not have any detectable levels of methylation in peripheral blood DNA. These tumours showed no *BRCA1 *methylation using MethyLight or MS-HRM, indicating that methylation of *BRCA1 *in the peripheral blood corresponds to tumour methylation.

The peripheral blood leukocyte DNA of seven age-matched normal controls was examined for *BRCA1 *methylation for comparison to the breast cancer patients. Six of the seven control samples did not have any detectable *BRCA1 *methylation by MethyLight or MS-HRM. One sample produced a methylated signal for two of three replicates by MethyLight only, estimated at the level of 0.1% relative to the 100% methylated control.

It is pertinent to consider the region that was spanned by the primers in each of our assays (Figure 1). In all cases, we designed primers that would not amplify the highly homologous duplication of the *BRCA1 *promoter region and exons 1 and 2 [22]. The MS-HRM assay overlaid the proximal promoter region and assessed the CpGs at -37, -29, -21 and -19 according to the nomenclature of Rice *et al*. [24]. Previous authors have shown some methylation in this region, commonly at -37 and -29 in peripheral blood in individuals with and without breast cancer [24,25]. We observed methylation in only three individuals using both MS-HRM and MethyLight assays. The MethyLight assay, while overlapping this region (the 3' ends of each primer being at -37 and +27), would only give a positive result if there were methylation at the probe binding site covering CpGs at +14, +16 and +19. This indicates that the three peripheral blood DNAs with detectable methylation were all methylated over a wider region than just -37 and -29. Remarkably, all three patients had tumours that were methylated for *BRCA1 *as measured by both assays. The -37 and -29 CpG sites could possibly represent a seeding area where under conditions of epigenetic instability, methylation of this small region would spread to the entire promoter causing inactivation of the entire *BRCA1 *promoter region.

Detailed analysis of the digital HRM assay was also instructive. Whereas the region assessed included -37 and -29, it also included the CpGs at -21 and -19 that tend not to be methylated. When the individual sequences of the amplified alleles were examined, all but one showed methylation at all four sites.

## Discussion

In this study, we demonstrate the presence of *BRCA1 *promoter methylation in normal non-epithelial tissues of patients that developed breast cancer. The levels of *BRCA1 *methylation in the peripheral blood (and buccal mucosa for KCF3) were confirmed by three independent methods, MethyLight, MS-HRM and digital MS-HRM. The observed methylation is not due to disseminated breast cancer cells in the blood because these do not occur at sufficiently high levels to be detected by our assay. Chen *et al*. have also looked for *BRCA1 *methylation in *BRCA1 *and *BRCA2 *mutation-negative women with a family history of breast cancer [25]. They did not detect any methylation in 41 patients. Each of these patients had approximately 10 clones examined, which would have made it difficult to detect low numbers of fully methylated alleles especially if there was any PCR bias in the amplification prior to cloning. It should also be noted that these women were unselected for *BRCA1*-like features and that the *BRCA1 *methylation status of their tumours was unknown.

We observed one peripheral blood leukocyte DNA sample in our age-matched control panel that had an extremely low level of *BRCA1 *methylation. This low level of methylation was not detectable by the MS-HRM assay. However, this result does raise the question of whether this individual does have an elevated risk of developing breast cancer in the future.

Many other questions remain to be addressed in future research following the intriguing findings presented here. Our results raise the possibility that methylation and associated silencing of *BRCA1 *could represent a germline alteration that underlies some cases of familial breast cancer. DNA from the parents of individual KCF3 were not available, so we were unable to determine if this was an epimutation that may have been inherited. In retrospect, patients from cancer families may not be the best subjects to examine for somatic methylation because of the likely limited transmissibility through the germline [10].

It is unclear why the levels of methylation in the somatic tissues were not at the 50% level that we expected. An interesting parallel may be drawn to a recent study showing somatic methylation in a hereditary non-polyposis colorectal cancer family. About 10% of the *MSH2 *gene alleles that segregated with the disease were methylated in DNA from peripheral blood [26]. The alteration was stably inherited in numerous individuals with the disease haplotype. The inheritance was not of a methylated allele *per se*, but of a propensity to methylate that was presumably associated with the allelic sequence. Although a methylation propensity could explain the deviation seen from expected allelic methylation ratios in our results, upstream sequencing of KCF3 (data not shown) did not detect any possible variation that could account for this.

## Conclusion

Our findings represent a new paradigm for somatic methylation leading to disease predisposition. Rather than an allelic methylation as has previously been reported for *MLH1*, a mosaic level of *BRCA1 *methylation has been identified in the somatic tissues of some breast cancer patients. We believe it will be important to examine individuals with early onset or multifocal breast cancer for *BRCA1 *methylation lacking a strong family history. We consider that widespread somatic methylation of *BRCA1 *is an important and as yet unrecognised cause of some *BRCA1*-like cancers. Further studies will be required to determine the frequency and functional significance of widespread somatic *BRCA1 *methylation.

## Abbreviations

ER = oestrogen receptor; kConFab = Kathleen Cuningham Consortium for Research in Familial Breast Cancer; MS-HRM = methylation-sensitive high resolution melting; PR = progesterone receptor.

## Competing interests

AD is listed as an inventor on provisional patents for the methylation-sensitive high resolution melting methodology.

## Authors' contributions

CS performed the MethyLight assays and assisted in writing the paper. MK performed further MethyLight assays as well as normal and digital MS-HRM assays, confirmatory sequencing, and assisted in writing the paper. EMW participated in developing the MS-HRM assays and sequenced the promoter region for patient KCF3. kConFab collected and curated the samples from familial cancer patients. ML reviewed the pathology assays and assisted in writing the paper. AD conceived the study, developed the research plan, supervised the research and was responsible for the writing of the paper.
